# Supplementary material for: Anatomy and function of the vertebral column lymphatic network in mice
Source: Nat Commun. 2019 Oct 9;10:4594. doi: 10.1038/s41467-019-12568-w (PMC6785564; doi:10.1038/s41467-019-12568-w)
Supplement: Supplementary file 1 — Supplementary Information [file 41467_2019_12568_MOESM1_ESM.docx]

**Supplementary information**

**Anatomy and function of the vertebral column lymphatic network in mice**

***Jacob et al***

**
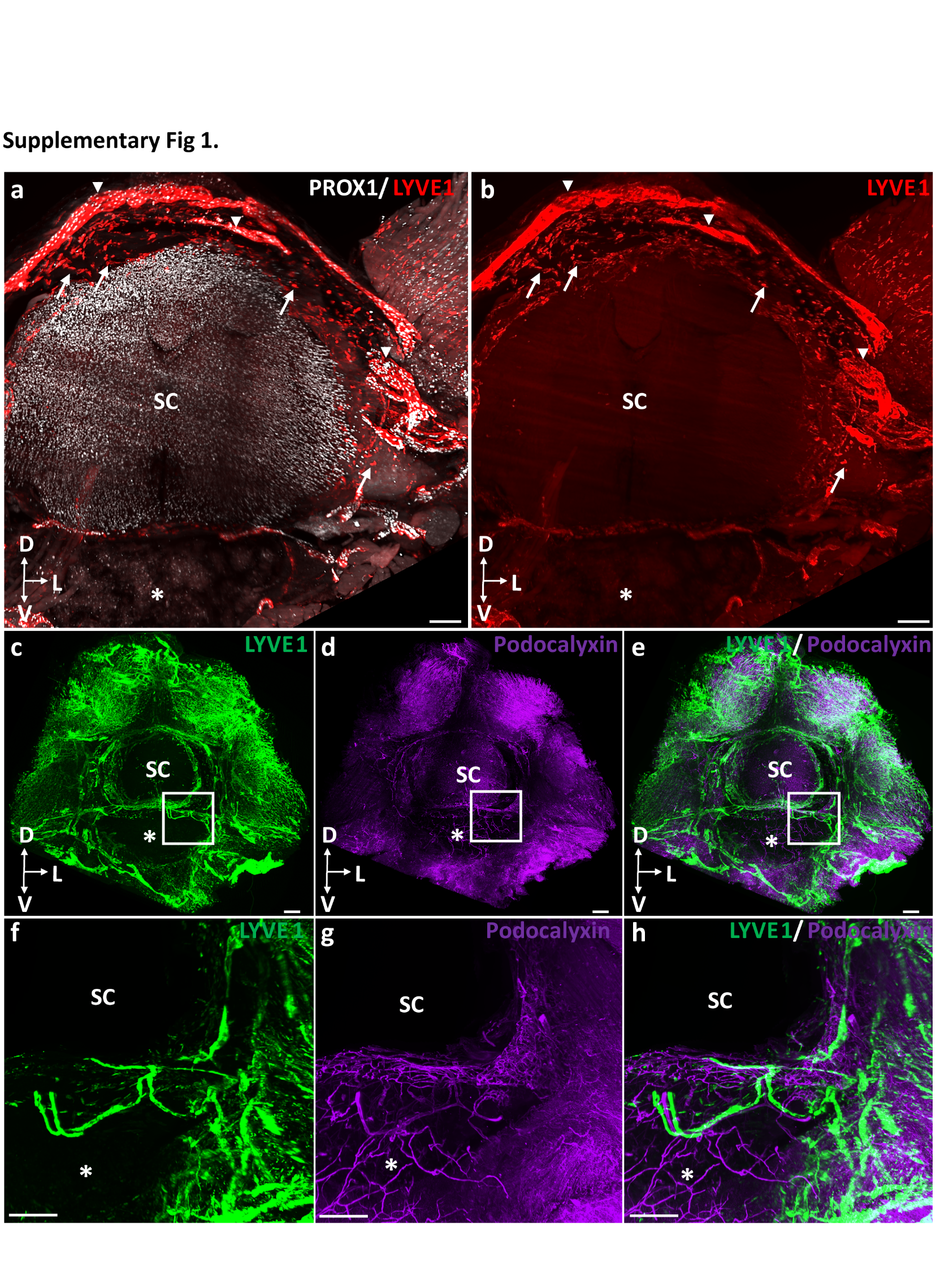
**

**Supplementary Fig 1.** LVs and blood vasculature in the thoracic vertebral column.

**a, b** PROX1 (white) /LYVE1 (red) double staining of a clarified thoracic vertebral column segment. Note: overlap of PROX1 and LYVE1 labeling in LVs (arrowheads); PROX1-labeling of SC oligodendrocytes **a** and LYVE1 staining of myeloid cells in meninges (arrows, **b**). **c**-**h** Double labeling of a clarified thoracic vertebral column segment with LYVE1 (green) and Podocalyxin (purple) to identify blood vessels. **f**-**h** : Magnifications of boxed areas in **c***-***e**. White asterisk: vertebral ventral body; SC: spinal cord. Scale bars: 300 µm **a**-**h**.

**
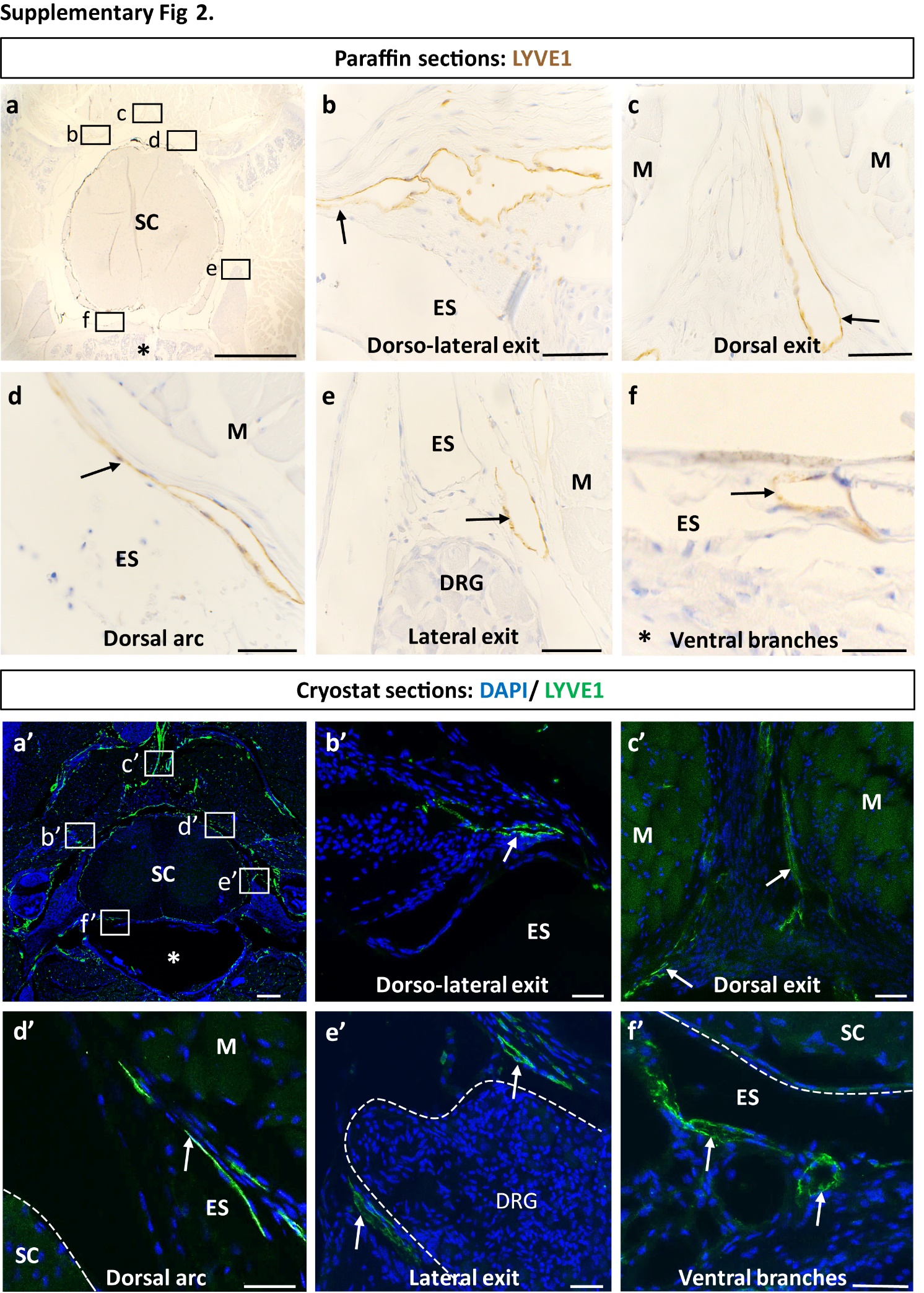
**

**Supplementary Fig 2.** vLV visualization on sections.

**a**-**f** Coronal paraffin sections **a-f** and cryosections **a’**-**f’** of thoracic vertebrae stained with anti-LYVE1 antibody (brown in **a**-**f** and green in **a’**-**f’**). DAPI is in blue **a’**-**f’**. Boxes indicate position of magnified images shown in **b**-**f** and **b’**-**f’**. vLVs exit the spinal vertebral canal via three routes: dorso-laterally (arrows in **b**, **b’**); dorsally (arrows in **c**, **c’**); and laterally along DRGs (arrows in **e**, **e’**). Inside the spinal vertebral canal: dorsal vLVs (arrows in **d**, **d’**) and ventral collaterals (arrows in **f, f’**). ES: epidural space, M: muscle, Asterisk: vertebral ventral body, SC: spinal cord. Scale bars: 1 mm **a**; 100 µm **b**-**f**; 400 µm **a’**; 50 µm **b’**-**f’**.

**
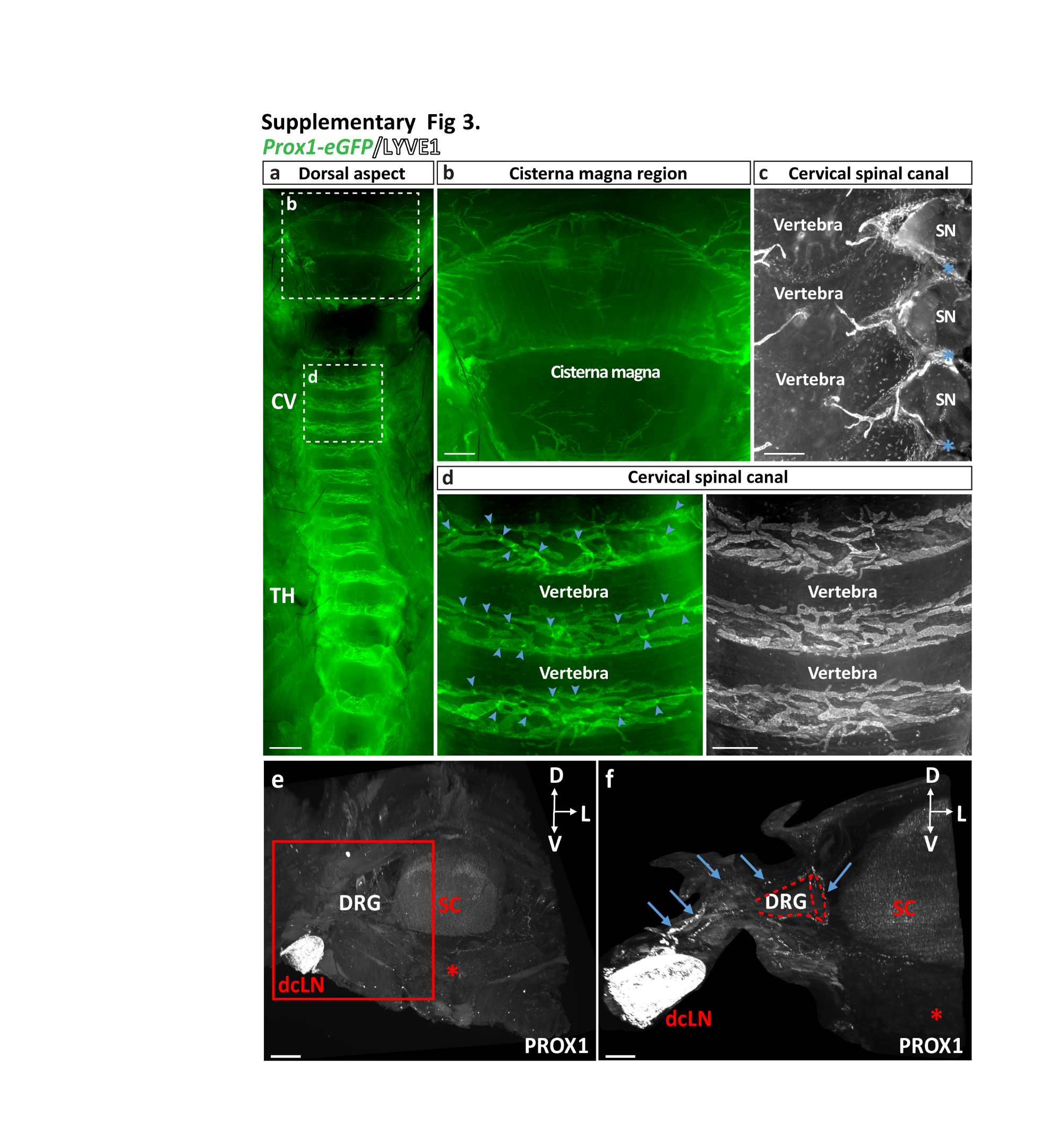
**

**Supplementary Fig 3.** vLV patterning varies between cervical and thoracic spine.

**a**-**d** Whole-mount immunostaining of *Prox1-eGFP* reporter mice showing lymphatic vasculature (PROX1 in green, LYVE1 in grey). **a** Dorsal lymphatic vasculature around cisterna magna and vertebrae in the cervical (CV) and thoracic (TH) region. **b** Close-up of lymphatic vessels around cisterna magna in close proximity with the LVs around the first cervical vertebra. **c** Side-view of the vertebral LVs connecting longitudinally with each other (marked with blue asterisks) and surrounding the spinal nerves (SN). **d** Close-up of vertebral lymphatic vessels in the dorsal aspect of the spine showing their localization mainly in intervertebral spaces. Blue arrowheads mark lymphatic valves. Data shown are representative of n = 3-6 segments/region. **e**, **f** PROX1 staining of the deep cervical LN (dcLN) in a cervical vertebral segment. Higher magnification view of boxed area in e shows the connection of dcLN with spinal root LVs (blue arrows) that appear to extend from the DRG lymphatic network f.. Asterisk: vertebral ventral body; SC: spinal cord. Scale bars: 1 mm **a**; 200 µm **b**, **c**; 400 µm **d**; 300 µm **e**, **f**.

**
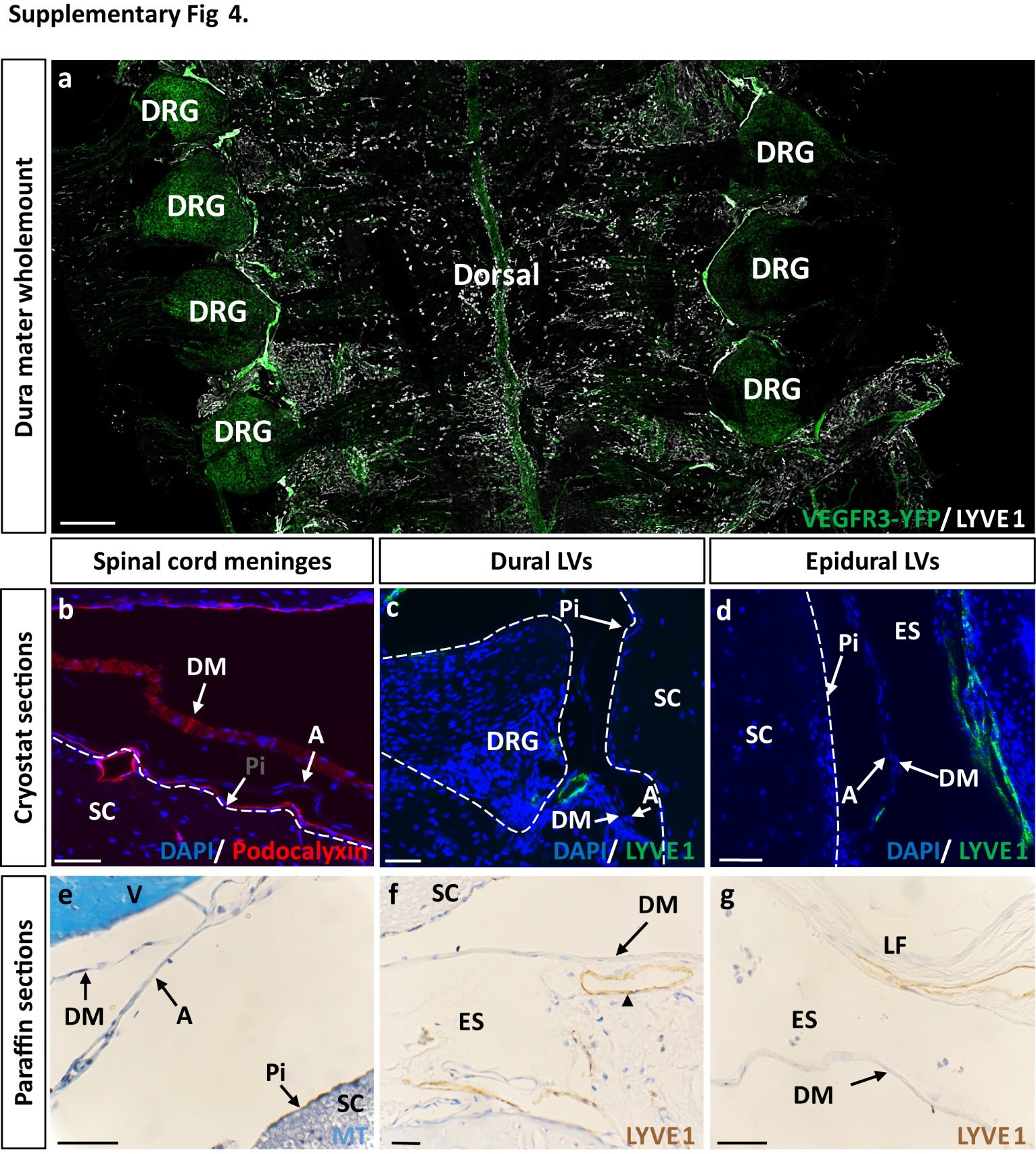
**

**Supplementary Fig 4.** Epidural and dural localization of vLVs.

**a** Dura mater whole mount of a *Vegfr3:YFP* reporter mouse immunostained with anti-LYVE1 (white) and anti-GFP (green) antibodies. Note that dural LVs are located only around the DRG and not in the dorsal region of meninges.

**b**-**d** Cryosections of different layers of thoracic spinal cord meninges labeled with anti-Podocalyxin antibody (red in **b**), anti-LYVE1 antibody (green in **c**, **d**), DAPI (blue in **c**, **d**). **b** illustrates the respective position of pial, arachnoid and dural meningeal membranes. **c**, **d** show the position of LYVE1^+^ vLVs relatively to the dura mater (**c**) and the epidural space (**d**).

**e**-**g** Paraffin sections of thoracic spinal cord meninges labeled with anti-LYVE1 antibody (brown in **f**, **g**). **e** Masson's trichrome staining (MT, blue) on paraffin sections shows the organization of meningeal layers (P: Pia mater, A: arachnoid, DM: dura mater). **f**, **g** Immunostaining with anti-LYVE1 antibody (brown, arrowhead) on paraffin sections. Note that vLVs arounds DRGs are located on the top of, or in contact with, the dura mater **f**. DRG: dorsal root ganglia, ES: epidural space, LF: ligamentum flavum, M: muscle, Asterisk: vertebral ventral body, SC: spinal cord, V: vertebrae. Scale bars: 250 µm **a**; 50 µm **b**-**d**; 80 µm **e**; 100 µm **f, g**.

**
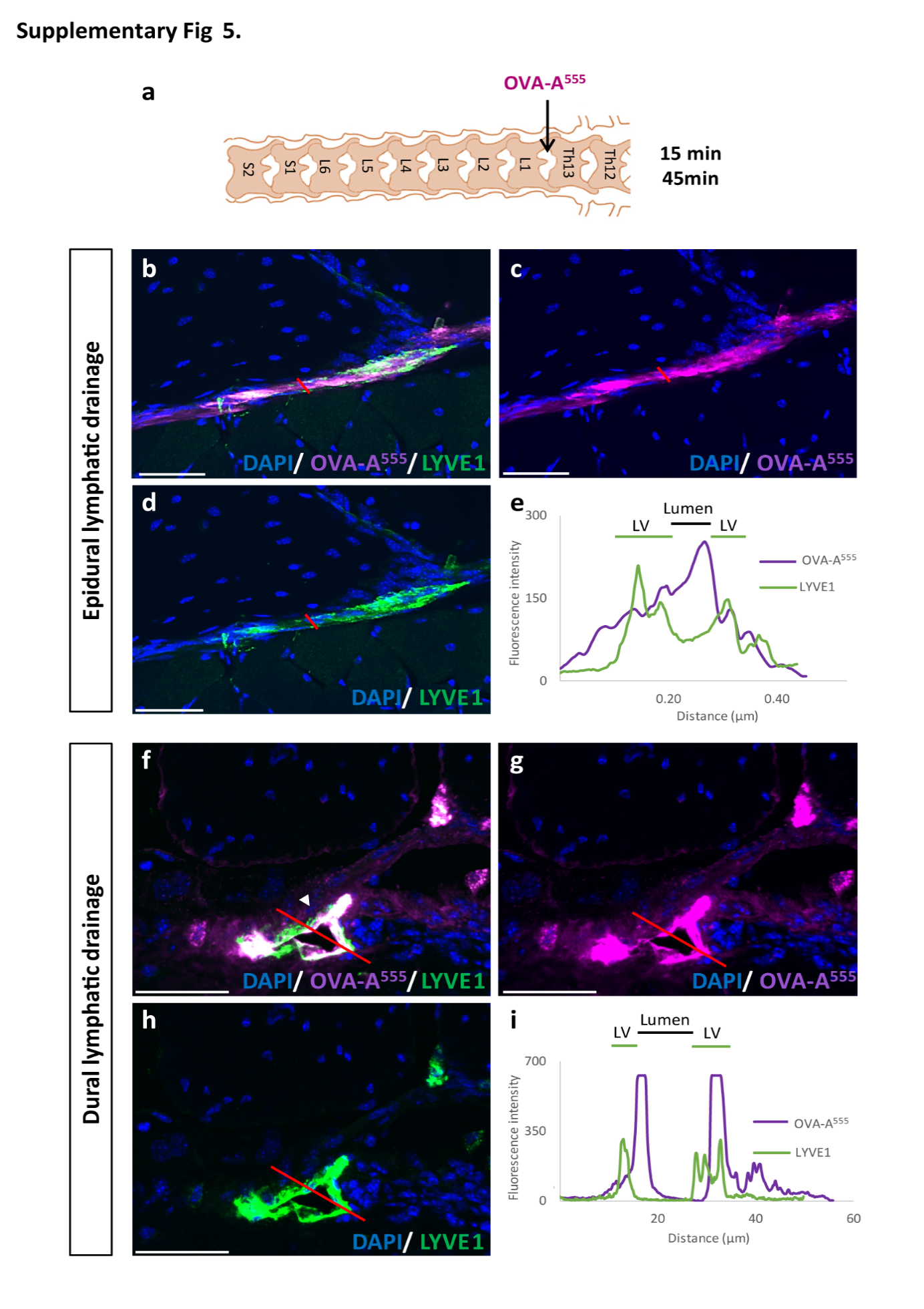
**

**Supplementary Fig 5.** Epidural and dural vertebral lymphatic drainage.

**a** Schematic representation of the experimental procedure: OVA-A^555^ is injected unilaterally in the thoraco-lumbar spinal cord parenchyme. 15 or 45 min after injection, OVA-A^555^ distribution pattern was visualized with a confocal microscope on spine cryosections labeled with anti-LYVE1 antibody. This figure is adapted from Fig 2c in Zhang Z-J. et al. Bio-protocol, 2016^53^.

**b**-**i** OVA-A^555^ (purple) colocalizes with lymphatic vessels (LYVE1^+^, green) in the dorsal epidural space **b**-**d**, as well as at the contact of dura mater (white arrowhead) **f**-**h**. DAPI nulear staining (blue). **e, i** Representative intensity profile plots for OVA-A^555^ tracer (purple) and LYVE1 (green) taken from a cross section of the images shown **b**-**d** and **f**-**h** indicating localization of OVA-A^555^ tracer within LYVE1^+^ vLVs. SC: spinal cord. Scale bars: 50 µm **b**-**h**.

**
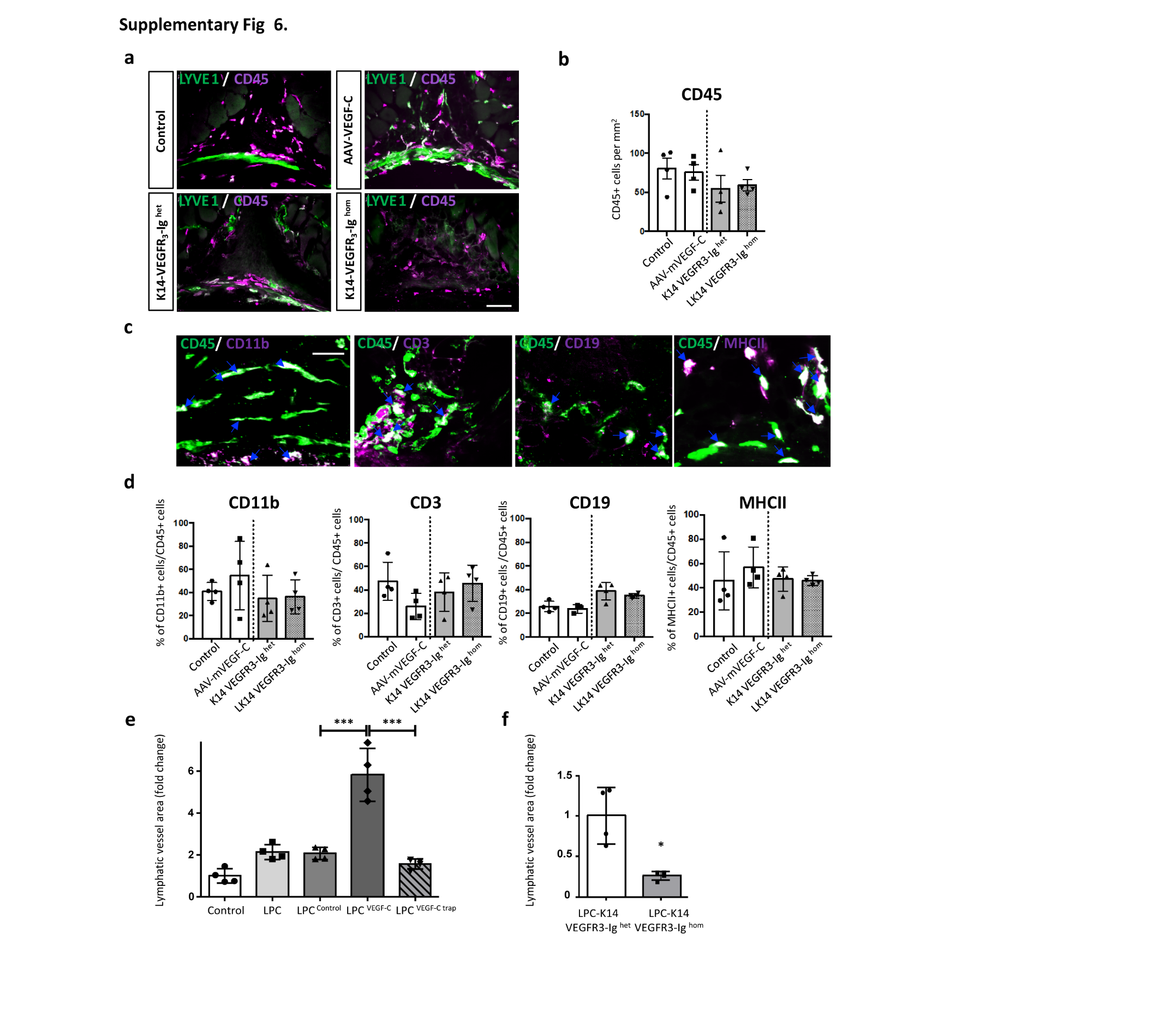
**

**Supplementary Fig 6.** Phenotype of epidural immune cells in control, AAV-mVEGF-C and K14-VEGFR3Ig mice.

**a** Dorsal epidural region of cervical vertebrae immunolabeled with anti-LYVE1 and -CD45 antibodies. Cervical vertebrae were isolated from WT control, LPC ^VEGF-C^, LPC-K14-VEGFR3-Ig^het^ or LPC-K14-VEGFR3-Ig^hom^ mice. **b** CD45^+^ leukocyte quantification in the epidural spaces illustrated in **a**. **c** Immunophenotyping of CD45^+^ leukocytes to detect antigens specific of myeloid cells (CD11b), T cells (CD3), B cells (CD19) and antigen-presenting cells (MHCII). **d** Histograms showing the ratio of myeloid cells, T cells, B cells and antigen-presenting cells among epidural leukocytes in control WT versus LPC ^VEGF-C^ and in LPC-K14-VEGFR3-Ig^het^ versus LPC-K14-VEGFR3-Ig^hom^ mice. n = 3-4 biologically independent mice/independent experiment, mean + /− SEM (error bar); Mann-Whitney U test. **e**, **f** Quantification of lymphatic vessel area (red stippled area in Fig 7i) after LPC-spinal cord injury in gain- and loss-of-mVEGF-C signaling mice (**e**) and in LPC-injured K14-VEGFR3-Ig^hom^ mice and -K14-VEGFR3-Ig^het^ (control) mice (**f**). n = 4 biologically independent mice/independent experiment and data show mean + /− SD (error bar) in (**e,** **f**); one-way ANOVA with Tukey’s multiple-comparisons test (**e**), and Mann Whitney U test (**f**); **p* < 0,05, ****p* < 0,001. Source data are provided as a Source Data file. Scale bars: 70 µm **a**; 35 µm **c**.

**
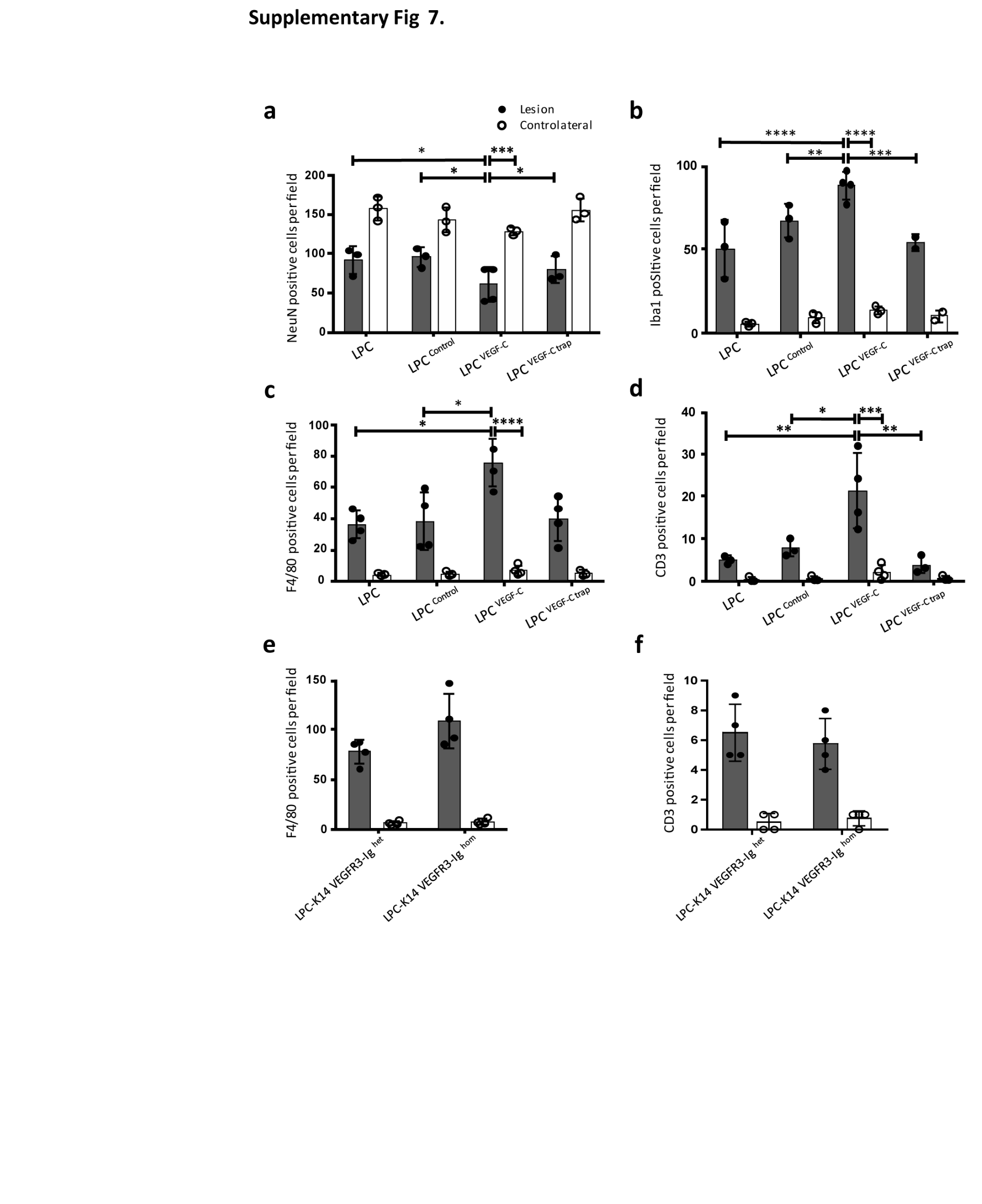
**

**Supplementary Fig 7.** VEGF-C increases T cell inflitration and tissue damage in mice with LPC-induced spinal cord injury.

**a**-**d** Quantifications of the indicated markers (NeuN (**a**), Iba1 (**b**), F4/80 (**c**) and CD3 (**d**)) on sections through the lesioned and contralateral sides of the spinal cord of LPC-injured mice. Mice were previously injected as indicated with AAV-mVEGFR3_4-7_-Ig (LPC ^control^), AAV-mVEGF-C (LPC ^VEGF-C^) or AAV-mVEGFR-3_1-3_-Ig (LPC ^VEGF-C trap^) in the lumbo-sacral region. **e**, **f** Histograms showing alterations of F4/80^+^ microglia/macrophage (**e**) and T cell (**f**) populations in the spinal cord parenchyme around the lesion, compared to the contralateral side, in LPC-K14-VEGFR3-Ig^het^ versus LPC-K14-VEGFR3-Ig^hom^ mice. n = 4 biologically independent mice/independent experiment, data represent mean + /− SD (error bar); two-way ANOVA with Tukey’s multiple-comparisons test, **p* < 0,05, ** *p*  < 0,01, *** *p*  < 0,001 and **** *p* < 0.0001. Source data are provided as a Source Data file.

| **Primary antibodies** | **Antibody dilution** | | | |
| --- | --- | --- | --- | --- |
|  | iDISCO+ | Paraffin sections | Cryostat sections | Whole mount |
| Rat anti–mouse podocalyxin (MAB1556; R&D Systems) | 1:2500 | * | * | * |
| Goat anti–human PROX1 (AF2727; R&D Systems) | 1:1200 | * | * | * |
| Rabbit anti–mouse LYVE-1 (11-034, AngioBio), | 1:800 | 1:100 | 1:800 | 1:800 |
| Polyclonal rabbit anti-mouse LYVE1^59^ | * | * | 1:1000 | 1:1000 |
| Rat anti-mouse LYVE1 (MAB2125, R&D Systems,) | * | * | 1:300 | 1:300 |
| Rabbbit anti-mouse Glut1 (07-1401, Milipore) | * | * | 1:100 | 1:100 |
| Goat anti-mouse CD45 (AF114; R&D Systems) | 1:2000 | * | 1:200 | 1:100 |
| Rat anti-mouse Cd11b (MA5-17857, Invitrogen) | * | * | * | 1:400 |
| Hamster anti-mouse CD3e (# 553058, BD Biosciences) | * | * | 1:100 | 1:100 |
| Rat anti-mouse CD3 (MAB4841; R&D Systems) | * | * | 1:100 | 1:100 |
| Rat anti-mouse CD19 (Alexa Fluor® 594 conjugated, BioLegend) | * | * | 1:50 | 1:50 |
| Rat anti-mouse MHCII (PE-conjugated, Invitrogen) | * | * | 1:800 | 1:800 |
| Rat anti-mouse F4/80 (MF48000, Invitrogen) | * | * | 1:100 | 1:100 |
| Rabbit anti-mouse Iba1 (019-19741, Wako) | * | * | 1:200 | 1:200 |
| Chicken anti-MBP (AB9348, Milipore) | * | * | 1:100 | 1:100 |
| Rabbit anti-mouse NeuN (GTX133127, GeneTex) | * | * | * | 1:100 |
| Rabbit anti–mouse Tyrosine Hydroxylase (T9237-13, US Biological) | 1:1500 | * | * | * |

**Supplementary Table 1.** List of primary antibodies
