# Supplementary material for: Anatomy and function of the vertebral column lymphatic network in mice
Source: Nat Commun. 2019 Oct 9;10:4594. doi: 10.1038/s41467-019-12568-w (PMC6785564; doi:10.1038/s41467-019-12568-w)
Supplement: Supplementary file 3 — Description of Additional Supplementary Files [file 41467_2019_12568_MOESM3_ESM.docx]

**Description of Additional Supplementary Files**

**File Name: Supplementary Movie 1**

**Description:** Lymphatic vasculature of a thoracic vertebral segment labeled with LYVE1 antibody. This 3D- movie corresponds to Fig. 1d, and shows the LYVE1 lymphatic vessel pattern around thoracic vertebrae. Note the segmented organization of the vertebral lymphatic network between vertebrae.

**File Name: Supplementary Movie 2**

**Description:** Lymphatic vasculature of a thoracic vertebral segment labeled with PROX1 antibody. This 3D-movie corresponds to Figs 1e, f, 2a-f, and shows the PROX1 lymphatic vessel pattern arround thoracic vertebrae. We carried out image segmentation for the vertebral lymphatic network, by removing the spinal cord and the peripheric lymphatic vessels.

**File Name: Supplementary Movie 3**

**Description:** Time-lapse z series of the PROX1-labeled lymphatic vasculature of a thoracic vertebral segment. This 2D-movie shows a LSFM transversal view of the PROX1 lymphatic vessel pattern around thoracic vertebrae, through the Z axis.

**File Name: Supplementary Movie 4**

**Description:** 3D-image segmentation of PROX1-labeled LVs of a thoracic vertebral segment. This 3D movie corresponds to Figs 1g, h and shows a thoracic vertebral segment immunolabel by PROX1 antibody. Each color defines the PROX1+ pattern of one intervertebral LV unit along two successive thoracic vertebrae (red, blue, green), as well as the peripheral lymphatic vasculature (white).

**File Name: Supplementary Movie 5**

**Description:** 3D-image segmentation of PROX1-labeled vLVs in meningeal and epidural layers around the cervical spinal cord. This 3D-movie corresponds to Figs 5a-d, and shows frontal images of the cervical vertebral column with color-coded layers: the arachnoid and dura mater in purple; the dura mater and epidural space in green; combination of color-coded layers showing the arachnoid in purple, the dura mater in white and the epidural space in green.

**File Name: Supplementary Movie 6**

**Description:** PROX1-labeled dural and epidural vLVs recapture injected with antiLYVE1 antibodies. This 3D-movie corresponds to Figs 6c-e, and shows the LYVE1 antibody uptake by epidural and dural lymphatic circuits, 45 min after l.s. injection. LYVE1 antibody (purple) colocalizes with LVs around DRG and with epidural LVs.

**File Name: Supplementary Movie 7**

**Description:** PROX1-labeled cervical vLVs following i.c.m. injection of AAV-mVEGF-C. This 3D-movie corresponds to Fig. 7c and shows the pattern of PROX1+ LVs (white) in cervical spine, one month after AAV-mVEGF-C injection. Image segmentation of the verbral lymphatic network allows to observe an important LV growth arround the spinal cord, induced by VEGF-C over expression.

**File Name: Supplementary Movie 8**

**Description:** PROX1-labeled lumbar vLVs at one week after spinal cord injury caused by focal LPC-injection. This 3D-movie corresponds to Fig. 7i, and shows the pattern of PROX1+ LVs (white) in the thoracic vertebral column, one week after LPC-injection. Lesion induices a strong vertebral LV growth around the spinal cord. Coronal section shows the LPC-injection site (without PROX1 staining) inside the spinal parenchyma.
