# Supplementary material for: Anatomy and function of the vertebral column lymphatic network in mice
Source: Nat Commun. 2019 Oct 9;10:4594. doi: 10.1038/s41467-019-12568-w (PMC6785564; doi:10.1038/s41467-019-12568-w)
Supplement: Supplementary file 12 — Reporting Summary [file 41467_2019_12568_MOESM12_ESM.pdf]

nature research

Corresponding author(s): Thomas Jean-Léon

Last updated by author(s): Aug 22, 2019

Reporting Summary

Nature Research wishes to improve the reproducibility of the work that we publish. This form provides structure for consistency and transparency in reporting. For further information on Nature Research policies, see Authors & Referees and the Editorial Policy Checklist.

Statistics

For all statistical analyses, confirm that the following items are present in the figure legend, table legend, main text, or Methods section.

n/a	Confirmed	
☐	☒	The exact sample size (n) for each experimental group/condition, given as a discrete number and unit of measurement
☐	☒	A statement on whether measurements were taken from distinct samples or whether the same sample was measured repeatedly
☐	☒	The statistical test(s) used AND whether they are one- or two-sided<br>*Only common tests should be described solely by name; describe more complex techniques in the Methods section.*
☒	☐	A description of all covariates tested
☐	☒	A description of any assumptions or corrections, such as tests of normality and adjustment for multiple comparisons
☐	☒	A full description of the statistical parameters including central tendency (e.g. means) or other basic estimates (e.g. regression coefficient) AND variation (e.g. standard deviation) or associated estimates of uncertainty (e.g. confidence intervals)
☒	☐	For null hypothesis testing, the test statistic (e.g. F, t, r) with confidence intervals, effect sizes, degrees of freedom and P value noted<br>*Give P values as exact values whenever suitable.*
☒	☐	For Bayesian analysis, information on the choice of priors and Markov chain Monte Carlo settings
☒	☐	For hierarchical and complex designs, identification of the appropriate level for tests and full reporting of outcomes
☒	☐	Estimates of effect sizes (e.g. Cohen's d, Pearson's r), indicating how they were calculated

Our web collection on statistics for biologists contains articles on many of the points above.

Software and code

Policy information about availability of computer code

Data collection	Axiovison, Zen, Leica Application Suite, ImSpector Software
Data analysis	Imaris software, ImageJ

For manuscripts utilizing custom algorithms or software that are central to the research but not yet described in published literature, software must be made available to editors/reviewers. We strongly encourage code deposition in a community repository (e.g. GitHub). See the Nature Research guidelines for submitting code & software for further information.

Data

Policy information about availability of data

All manuscripts must include a data availability statement. This statement should provide the following information, where applicable:

- Accession codes, unique identifiers, or web links for publicly available datasets
- A list of figures that have associated raw data
- A description of any restrictions on data availability

The source data underlying Figs. 7j, k and 8f-h as well as Supplementary Figs. 6b, 6d-f and 7a-f are provided as a Source Data file. All data supporting the findings of this study are available from the corresponding authors upon reasonable request.

Field-specific reporting

Please select the one below that is the best fit for your research. If you are not sure, read the appropriate sections before making your selection.

☒ Life sciences ☐ Behavioural & social sciences ☐ Ecological, evolutionary & environmental sciences

For a reference copy of the document with all sections, see nature.com/documents/nr-reporting-summary-flat.pdf

Life sciences study design

All studies must disclose on these points even when the disclosure is negative.

Sample size	The sample size of four animals for each group was determined according to sample number required to ANOVA (one or two-way) or Mann Whitney U test statistical analysis.
Data exclusions	No data were excluded from the analyses. All animals with proper experimental conditions were included in the analysis.
Replication	All the experiences were repeat almost 3 times, only LPC lesion experiments was done 2 times
Randomization	No randomization.
Blinding	All the analysis was done in double-blinded.

Reporting for specific materials, systems and methods

We require information from authors about some types of materials, experimental systems and methods used in many studies. Here, indicate whether each material, system or method listed is relevant to your study. If you are not sure if a list item applies to your research, read the appropriate section before selecting a response.

Materials & experimental systems

n/a	Involved in the study	
☐	☒	Antibodies
☒	☐	Eukaryotic cell lines
☒	☐	Palaeontology
☐	☒	Animals and other organisms
☒	☐	Human research participants
☒	☐	Clinical data

Methods

n/a	Involved in the study	
☒	☐	ChIP-seq
☒	☐	Flow cytometry
☒	☐	MRI-based neuroimaging

Antibodies

Antibodies used

iDISCO staining:
Goat anti-mouse CD45 (1:2000, AF114; R&D Systems)
Rabbit anti–mouse LYVE-1 (1:800;11-034, AngioBio)
Rat anti–mouse Podocalyxin (1:2500 MAB1556; R&D Systems)
Goat anti–human PROX1 (1:1200, AF2727; R&D Systems)
Rabbit anti–mouse Tyrosine Hydroxylase (1:1500, T9237-13, United States Biological)
Staining of vertebral cannal whole mounts and spinal cord sections:
Rabbit anti–mouse LYVE-1 (1:800;11-034, AngioBio)
Goat anti-mouse CD45 (1:10 AF114; R&D Systems)
Hamster anti-mouse CD3e (1:100, # 553058, BD Biosciences) or Rat anti-mouse CD3 (1:100 MAB4841; R&D Systems)
Rat anti-mouse MHCII (1:800, PE-conjugated, Invitrogen)
Rat anti-mouse CD19 (1:50, Alexa Fluor® 594 conjugated, BioLegend)
Rat anti-mouse Cd11b (1:400, MA5-17857, Invitrogen)
Rat anti-mouse F4/80 (1:100, MF48000, Invitrogen)
Chicken anti-MBP (1:100, AB9348, Milipore)
Rabbit anti-mouse NeuN (1:100, GTX133127, GeneTex)
Rabbit anti-mouse Iba1 (1:200, 019-19741, Wako)
Rabbbit anti-mouse Glut1 (1:100, 07-1401, Milipore).

Validation

Each antibody was validated in immunochemistry based on standard validation protocols.

Animals and other organisms

Policy information about studies involving animals; ARRIVE guidelines recommended for reporting animal research

Laboratory animals	Vegfr3:YFP lymphatic reporter mice line; K14-VEGFR3-Ig mice56; Prox1-eGFP lymphatic reporter mice line.
Wild animals	C57BL/6J mice,mal, 2-3months old
Field-collected samples	The study did not involve samples collected from the field.

Ethics oversight

All in vivo procedures used in this study complied with all relevant ethical regulations for animal testing and research, in accordance to the European Community for experimental animal use guidelines (L358-86/609EEC). The study received ethical approval by the Ethical Committee of INSERM (n°2016110111126651) and the Institutional Animal Care and Use Committee of ICM (Institut du Cerveau et de la Moelle épinière).

Note that full information on the approval of the study protocol must also be provided in the manuscript.
